# Design and Haemodynamic Analysis of a Novel Anchoring System for Central Venous Pressure Measurement

**DOI:** 10.3390/s22218552

**Published:** 2022-11-06

**Authors:** Tejaswini Manavi, Masooma Ijaz, Helen O’Grady, Michael Nagy, Jerson Martina, Ciaran Finucane, Faisal Sharif, Haroon Zafar

**Affiliations:** 1 Cardiovascular Research & Innovation Centre, University of Galway, H91 TK33 Galway, Ireland; 2 Lambe Institute for Translational Research, School of Medicine, University of Galway, H91 TK33 Galway, Ireland; 3 Endotronix, Inc., Lisle, IL 60532, USA; 4 Department of Medical Physics and Bioengineering, Mercer’s Institute for Successful Ageing, St James’s Hospital Dublin, D08 NHY1 Dublin, Ireland; 5 Department of Cardiology, University Hospital Galway, H91 YR71 Galway, Ireland; 6 BioInnovate, H91 TK33 Galway, Ireland; 7 College of Science and Engineering, University of Galway, H91 TK33 Galway, Ireland

**Keywords:** central venous pressure, heart failure, remote patient management, wireless pressure sensor, inferior vena cava, anchoring system, haemodynamic analysis, bench testing, computational fluid dynamics

## Abstract

Background/Objective: In recent years, treatment of heart failure patients has proved to benefit from implantation of pressure sensors in the pulmonary artery (PA). While longitudinal measurement of PA pressure profoundly improves a clinician’s ability to manage HF, the full potential of central venous pressure as a clinical tool has yet to be unlocked. Central venous pressure serves as a surrogate for the right atrial pressure, and thus could potentially predict a wider range of heart failure conditions. However, it is unclear if current sensor anchoring methods, designed for the PA, are suitable to hold pressure sensors safely in the inferior vena cava. The purpose of this study was to design an anchoring system for accurate apposition in inferior vena cava and evaluate whether it is a potential site for central venous pressure measurement. Materials and Methods: A location inferior to the renal veins was selected as an optimal site based on a CT scan analysis. Three anchor designs, a 10-strut anchor, and 5-struts with and without loops, were tested on a custom-made silicone bench model of Vena Cava targeting the infra-renal vena cava. The model was connected to a pulsatile pump system and a heated water bath that constituted an in-vitro simulation unit. Delivery of the inferior vena cava implant was accomplished using a preloaded introducer and a dilator as a push rod to deploy the device at the target area. The anchors were subjected to manual compression tests to evaluate their stability against dislodgement. Computational Fluid Dynamics (CFD) analysis was completed to characterize blood flow in the anchor’s environment using pressure-based transient solver. Any potential recirculation zones or disturbances in the blood flow caused by the struts were identified. Results: We demonstrated successful anchorage and deployment of the 10-strut anchor in the Vena Cava bench model. The 10-strut anchor remained stable during several compression attempts as compared with the other two 5-strut anchor designs. The 10-strut design provided the maximum number of contact points with the vessel in a circular layout and was less susceptible to movement or dislodgement during compression tests. Furthermore, the CFD simulation provided haemodynamic analysis of the optimum 10-strut anchor design. Conclusions: This study successfully demonstrated the design and deployment of an inferior vena cava anchoring system in a bench test model. The 10-strut anchor is an optimal design as compared with the two other 5-strut designs; however, substantial in-vivo experiments are required to validate the safety and accuracy of such implants. The CFD simulation enabled better understanding of the haemodynamic parameters and any disturbances in the blood flow due to the presence of the anchor. The ability to place a sensor technology in the vena cava could provide a simple and minimally invasive approach for heart failure patients.

## 1. Introduction

Heart Failure (HF) affects an estimated population of 26 million people worldwide. It is associated with significant hospitalization in the United States and Europe, with over 1 million admissions per year reported in both the countries [1,2,3]. Within the USA alone, the prevalence of HF is 5.7 million with 870,000 cases diagnosed every year [3]. In Ireland, HF affects approximately 90 k people. Most of these present without symptoms despite having a significant underlying cardiac function impairment, and are therefore at a higher risk of future development of this condition [4,5].

With the incidence of HF on the rise, the notion of designing and developing a simple, valid, and reliable system to monitor symptoms or progression of HF is widely acknowledged [6,7,8]. There is an increased appetite for home-based or remote monitoring technologies following COVID-19, especially for patients with HF. Remote patient management (RPM) could provide an early indication of HF decompensation events and could potentially allow for optimization of patient therapy to prevent HF hospital visits [8].

### 1.1. Central Venous Pressure (CVP): A Potential Metric for HF Monitoring

CVP and its surrogate right atrial pressure (RAP) could provide essential diagnostic information for the treatment of pathologies as diverse as right-sided and left-sided heart failure, chronic kidney disease, acute kidney injury, pulmonary hypertension, liver disease, and most importantly, systemic congestion [9]. An increase in left ventricular diastolic pressure (LVDP) associated with signs and symptoms of HF such as dyspnoea and oedema is an indicator of clinical congestion. However, there are a series of pathophysiologic changes that precede clinical or systemic congestion. These changes occur during haemodynamic congestion, referred to an elevation of LVDP in HF patients without overt signs and symptoms [9]. Abnormal left ventricular function may be characterized by an increase in LVDP, resulting in further increase in the left atrial and ventricular diastolic pressures followed by mitral regurgitation. These events induce haemodynamic congestion characterized by an increase in pulmonary capillary wedge pressure (PCWP) and pulmonary artery pressure (PAP). Elevation in PAP further results to an increase in the right atrial and ventricular pressures associated with signs of jugular venous distension (JVD) and oedema [10,11,12,13,14]. Therefore, systemic congestion could be the ‘tip of the iceberg’ of haemodynamic derangements that precede symptoms. Elevation in right heart pressures may also contribute to the cardio-renal syndrome associated with reduction of perfusion-gradient across the kidneys [15,16,17,18].

Controlling congestion is the key challenge to heart failure management, which helps stabilize symptoms and sudden health deterioration eventually resulting in death [17]. Clinical congestion is an important target for therapy, but is associated with poor prognosis [19,20]. Future implantable HF monitoring systems need to incorporate central venous pressure as a potential haemodynamic parameter to track systemic congestion or the events that precede its symptoms.

### 1.2. Modes of CVP Measurement

Central venous catheterization remains the gold standard for CVP measurement. Although regarded as the most accurate and effective method, it is a time consuming invasive procedure, and associated with potential complications involving infections, catheter-induced thrombosis, and arterial puncture [21,22,23,24].

An alternative method to estimate CVP is through assessment of peripheral venous pressure (PVP) by transducing a peripheral intravenous line. Such studies have yielded mixed results. Correlations between PVP and CVP need to be studied specifically in a heart failure population [25,26].

Another non-invasive technique is the near-infrared spectroscopy used to detect jugular venous pressure (JVP) in the neck. This system uses a single wavelength LED and photodetector to measure JVP, and requires the patient to lie down at an angle where the JVP pulse aligns with the infrared sensor. Although this method yields similar results to those measured invasively, it is not used routinely in a clinical setting [27].

A study by Marcelli et al. introduced a non-invasive device for CVP assessment designed for use in hospitals, offices, and home. This device operates on the principle of venous occlusive plethysmography, extensively used for other applications in a clinical setting. Limitations of this technique requires the patients to be mobilized with the cuffs connected to a graphical user interface system [28]. Moreover, it requires clinical validation in a certain group of HF patients with multiple comorbidities to determine the accuracy of measurements, as any pathophysiologic changes affecting the pressures between left ventricle and right atrium can affect the measured CVP. Other non-invasive methods are based on ultrasounds which display limited accuracy, are operator dependent, and subjective to interpretive error [29,30]. A more continuous form of monitoring is required for longitudinal measurements of CVP in real-time, which is now possible through modern sensor development.

Currently, to the author’s knowledge, there is limited research focusing on implantable or wireless monitoring techniques for continuous real-time measurements of CVP. This study aimed to address the following unmet needs:

- CVP measurements targeting inferior vena cava (IVC) as a potential site of interest remains unexplored.
- Current sensor anchoring methods, which are effective for other arteries e.g., pulmonary artery, may fail to hold the sensor safely in place in the physically more challenging environment of the IVC (as it is a thin and compliant vessel with a smaller diameter as compared with the arteries).
- Significant physical and functional adaptation of existing haemodynamic sensors is required for a safe and effective implant in the IVC.

#### 1.2.1. Importance of Anchoring in the IVC

Haemodynamic IVC monitoring presents a challenge arising from the physiology of IVC. IVC is relatively a compliant vessel as compared with others and is prone to easy distortion due to forces applied by implants. This may adversely affect the performance and the precise monitoring capability of IVC implants. There is a requirement of a reliable and affordable wireless monitoring system, particularly in the field of heart failure monitoring. A unique challenge for implants in the IVC is its asymmetrical shape and dynamics during collapse and expansion. IVC tends to collapse in the anterior–posterior direction, moving from a circular shape to a flat, oval cross-section, resulting in a change in blood volumes [31]. An implant with a large radial force can change the configuration of IVC vessel cavity and affect the collapsible performance of IVC. This may affect the venous blood return causing a change in the haemodynamic area around the venous implant, resulting in the formation of thrombosis [32].

Design and optimization of an anchoring system in the IVC for a safe long-term implant with minimal or no-risk is an unmet clinical need. Some studies have emphasized the anchoring mechanism of vena cava filters and stents, indicating the requirement for additional post-implant procedures to secure the stent or filter from migrating to the right heart. More than 71% of patients with intravascular foreign bodies experience major complications such as cardiac arrhythmias, sepsis, and cardiac tamponade [33]. Post-implant procedural complications such as stent interlocking or extension may cause contralateral iliac vein obstruction or even deep vein thrombosis [34,35,36]. A robust anchoring system that will minimize or eliminate the need for post-implant procedures in the IVC should be developed.

Recommendations to improve anchoring include oversizing by selecting a stent diameter greater than the diameter of the vein, interlocking stent techniques and pull-through wire system that would facilitate a safe and precise placement of the device [33,37]. Physicians do not recommend routine anchoring of stents. However, the anchoring techniques are commonly implemented post stent implantation when in-stent restenosis and venous outflow obstruction are noted [34,35,36]. This implies the need for further optimization and development of anchoring techniques to prevent implant-related complications earlier than when they become emergent at a later stage.

#### 1.2.2. Design Challenges of Existing Implants in the Venous System

Over the last few decades, two types of implanted devices have targeted the IVC; the vena cava filters and venous stents. IVC filters have gained popularity to prevent acute pulmonary embolism (PE) in patients with deep vein thrombosis (DVT). Although the vena cava filters proved to have a significant benefit on mortality in unstable patients [38], several long-term complications have been reported, including IVC occlusion rate of up to 30% [39,40] and IVC penetration rate ranging between 0% and 86% [41,42].

Venous stents are gaining popularity in the field of interventional medicine and serve as a new treatment paradigm for patients with chronic venous obstruction. Some major technical limitations related to the design of Wallstents (Boston Scientific, Marlborough MA, United States) have been reported. The first shortcoming is the weaker edge of stent that is prone to occlusion or collapse when placed in a diseased or compressed iliac vein [43]. Unsuccessful deployment and lack of precision due to the braided design matrix account for the second shortcoming of such stents.

Learnings from previous implants (filters and stents) in IVC highlight the requirement of a safe anchoring system that would overcome limitations such as IVC penetration, embolization, occlusion, and device migration.

In this study, we introduced a novel anchoring system compatible with existing sensor technology in the IVC for the purpose of non-invasive CVP measurement. We tested the feasibility and ease of implantation of the novel anchor in a suitable bench test model. Investigation of the haemodynamic parameters of the anchor was performed using CFD analysis, simulating blood flow inside a 3D IVC model with the anchor.

## 2. Methods and Materials

**Figure 1:**
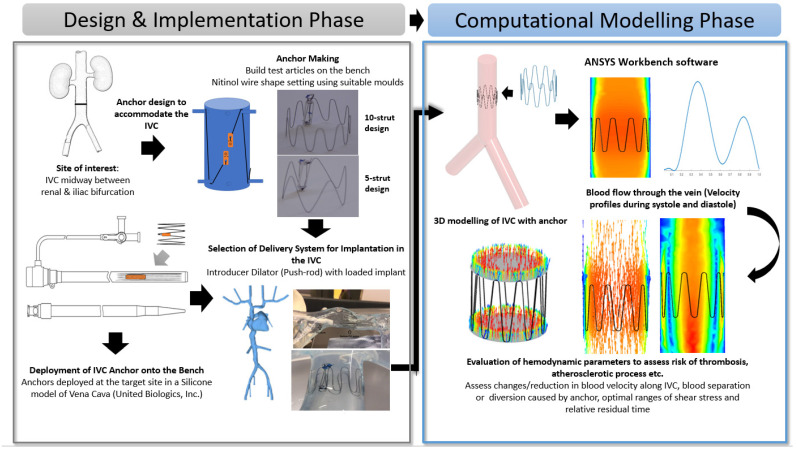
Process flow illustrating the Anchor Design and Implementation Phase (Bench-Top Simulation) and Computational Modelling Phase (Introduction to CFD modeling and its parameters).

This section details the design implementation of the anchor on the bench and its numerical analysis using CFD. The process flow (Figure 1) provides an illustrative overview of the design and selection of the anchor and the evaluation of its haemodynamic parameters through CFD modelling.

### 2.1. Selection of Optimal Site for Accommodation of the Anchor

**Figure 2:**
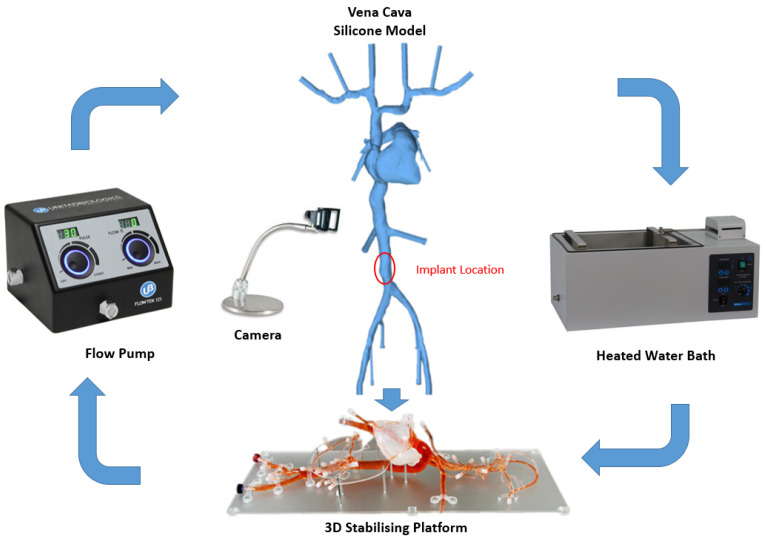
Bench top model plan and peripherals (silicone model of Vena Cava, stabilizing platform and flow pump, United Biologics, Inc., Irvine, CA, USA).

IVC is considered as an optimal site for implantation since it presents a straight tube and provides a relatively easy access point when compared with the superior veins [44]. According to a previous study conducted by Manavi et al., a location midway between the renal veins and iliac bifurcation serves as a potential site for implantation of a haemodynamic sensor [44]. This long straight portion of the vessel has a potential for good anchorage as compared with the branched vessels in the superior vena cava. The area between the renals and iliacs allow for a less complex anchor design in the straight vessel as compared with the supra-renal IVC that tapers outwards and lies in proximity of the portal or hepatic veins. The optimal location in the IVC and the bench top set-up is illustrated in Figure 2.

### 2.2. Anchor Fabrication

A novel anchor was designed to accommodate the specific constraints of the target site in the IVC. The design of this anchor is based on pre-existing arterial stents but simplified to a single thread of ten struts arranged cylindrically.

The nitinol anchor wires (Fort Wayne Metals, Fort Wayne, IN, USA) have an austenite final temperature below room and body temperatures. This results in anchor wires maintaining their super-elastic properties under normal use cases and not utilizing the temperature-dependent shape memory return to perform their intended function, which is common to other nitinol wire applications.

The straight nitinol wires were shape set using suitable fixtures or moulds (Riteway Engineering Ltd., Galway, Ireland). This mold allows anchor struts to be arranged cylindrically with termination points facing away from the cylindrical mould that allows attachment to the sensor body. The anchor wires with the fixture were subjected to heat treatment at 550 °C in a box furnace (Thermo Scientific^TM^ BF51828C1, Waltham, MA, USA). The dwell time range to shape set anchors was set to 16 min, 15 s ± 10 s. The moulds were quenched in a water bath (35 °C) for 30 s. Anchors were visually inspected to ensure the desired shape was set in the wire.

**Figure 3:**
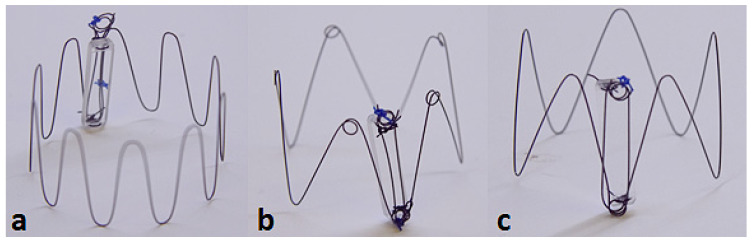
Three candidate anchor designs: 10-strut (**a**); 5 strut with loops (**b**); 5-strut without loops (**c**) attached to Dummy Sensor Bodies.

Three candidate anchor designs were fabricated that vary in the number of struts and the presence of loops:

- 10-strut anchor design attached to a dummy sensor body (Figure 3a).
- 5-strut anchor design with loops, designed to improve the outward radial force (ORF) and prevent the risk of dislodgement post deployment (Figure 3b).
- 5-strut anchor design without loops (Figure 3c).

Dummy glass sensor bodies (20 mm × 4 mm × 2 mm) were used for the purpose of bench deployments to evaluate anchorage and deployment accuracy. Attachment of the anchor to the dummy sensor body was established using nylon sutures. The termination points of the anchor constitutes a loop (Figure 3) intended for suture placement. The tie-down or attachment process involves a suture tied down to the looped-part of the anchor connecting the sensor body at its termination points (two holes each located at the top and bottom edge of sensor body) enabling firm attachment.

### 2.3. Bench Test Procedure

The bench test model is a silicone model of the Vena Cava (United Biologics, Inc., Irvine, CA, USA) vasculature with superior and inferior veins, right heart, and coronary sinus.

**Figure 4:**
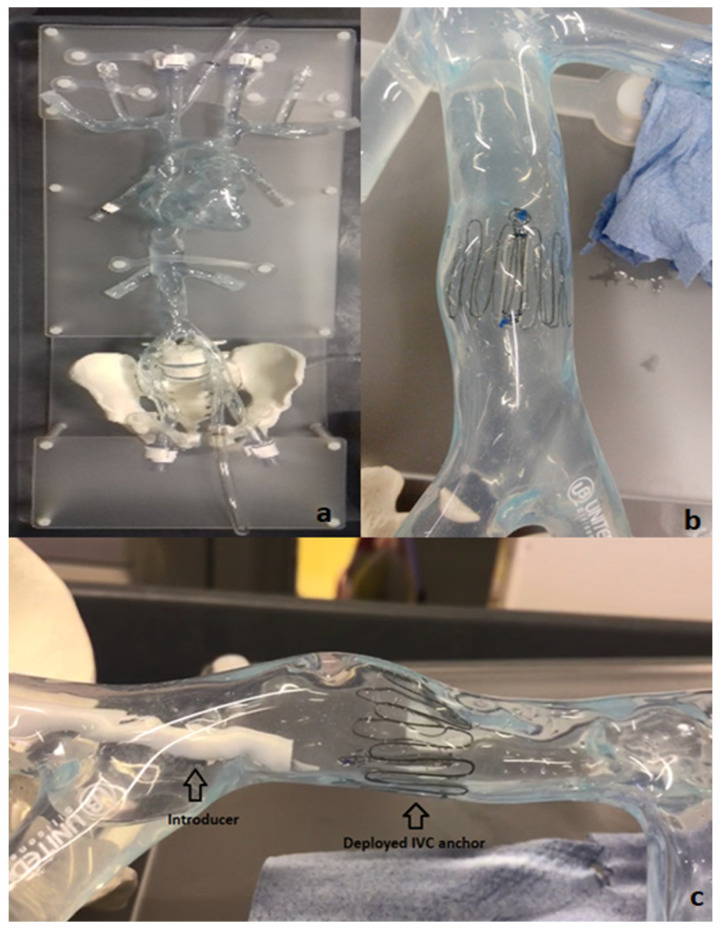
Bench model of Vena Cava (**a**); 10-strut anchor deployed at the target site in the Vena Cava model (**b**); deployment of the anchor in the bench model using an introducer–dilator as the delivery system (**c**).

To evaluate the deployment of the novel anchor design in the IVC, a bench top stand was set up which included a 3D printed model of the relevant anatomy connected to a pulsatile pump system (Flowtek 125, United Biologics, Inc., Irvine, CA, USA) (Figure 4a). The deployment of the anchor and sensor system was evaluated using this set-up (Figure 4b). The suitability of the new anchor design in the target anatomy of the 3D model was determined and any potential modifications to the design were identified after deployments. In addition, a new delivery system involving an introducer sheath and dilator technique was evaluated for use in the bench model (Figure 4c).

Figure 2 below shows the Bench Model system used to test anchors. The pump simulated the regular pulse and allowed steady flow in the model. This set-up was connected to a heated water bath (Fisher Scientific, Dublin Ireland) (37 °C) to simulate body temperature. The addition of 0.6% baby shampoo to water within the bench model enabled a lubricated flow similar to blood.

A set of Introducers (Cook Medical, Limerick, Ireland) ranging from 14 Fr–22 Fr were used to test suitability for loading and deploying the IVC anchors on the bench model. Various combinations of introducers and anchors were tested on the bench to guide selection of an optimal delivery system. The anchors were crimped and loaded at the tip of the introducer using an appropriate size Falcon tube. The objective for testing various range of introducers is to check the possibility of any kinks during anchor loading, any oversized introducer-anchor combinations, and to assess the ability of the anchor to move effortlessly within the introducer following manual push force from the dilator ensuring appropriate deployment.

**Figure 5:**
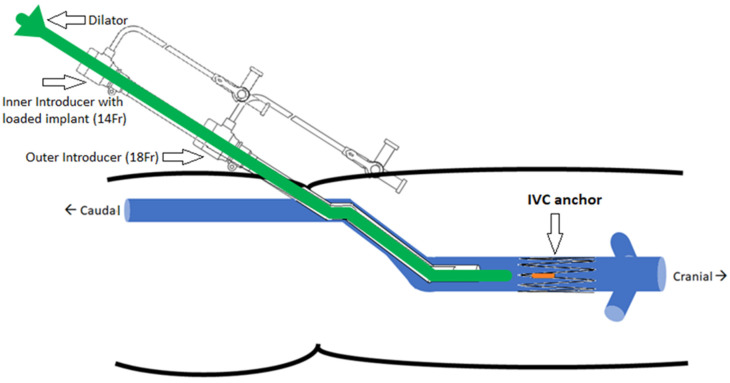
Implantation concept of the IVC anchor (double introducer push-rod concept).

The implantation concept is illustrated in Figure 5. The selected delivery system consists of two introducers: an outer one (18 Fr) used as a guide and placed at the target site and an inner one (14 Fr) with the loaded implant inserted through the outer introducer. The dilator acts as a push rod to deploy the device at the target area.

The opening or access site in the model is the right iliac vein secured with a haemostatic valve. The introducer and dilator were advanced through this valve to achieve implantation at the target location. The device was deployed by pushing the dilator rod through the introducer sheath to release the anchor with sensor body attached. After deployment, anchorage at the target location was inspected visually in the anatomical model. In the next step, collapse of the vessel was induced using external physical force or pinching of the model at the location of the device. The model was disturbed and compressed from all angles and along the length of the device. These compression and stress tests were performed to analyze deployment orientation and potential for device dislodgement.

Fifteen anchor deployments in total (five attempts per design) were executed on the bench. Accuracy of deployment and anchorage within the vessel were assessed for all the anchor designs. Manual compression tests were performed post deployment on each of the anchor designs to assess the risk of dislodgement, and evaluate if the designs could withstand vein collapsibility or dynamic force exerted on them. This has a potential clinical significance in heart failure/hypervolemic patients with a vena cava collapse of ≤50 percent [45]. It is important to consider the effects of percentage collapse of the vein during respiration cycles on implants in the IVC.

The criteria for successful deployment were determined as if the device was deployed at the desired location in the correct position with the anchor struts secured against the vessel wall and if the sensor body was in the desired position. Successful anchorage was determined if the sensor body and anchor remained in the position after several compression tests.

### 2.4. Device Design Parameters and CAD Modelling

**Figure 6:**
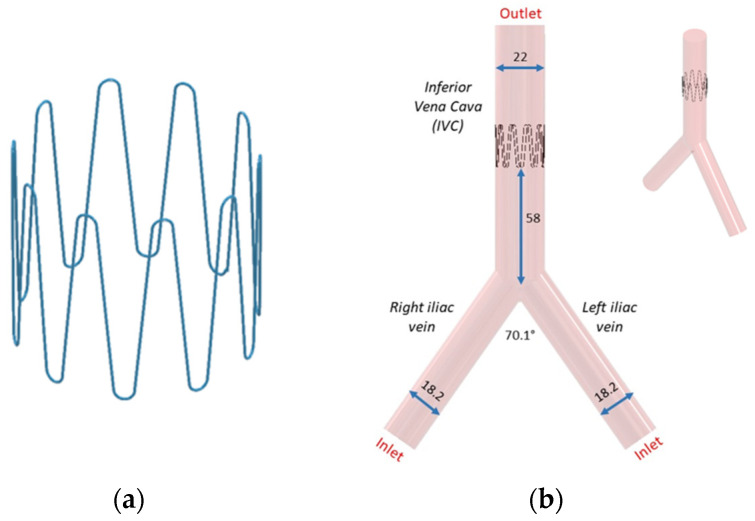
(**a**) Anchor and (**b**) its placement in the IVC. All dimensions are in mm.

Following bench test simulations, a CAD profile of the selected 10-stut anchor design (Figure 6a) and the IVC model (Figure 6b) was constructed. The 3D IVC model consists of blood flow from the right and left iliac vein. A 22 mm diameter 10-strut anchor was selected so that the anchor walls are in direct contact with the vein walls. The anchor is 20 mm in height and has 0.28 mm thick struts equidistant from each other. The anchor is positioned at 58 mm above the iliac bifurcation point (Figure 6b). The 3D IVC model dimensions correspond to the vena cava bench test model in Figure 4.

### 2.5. Computational Modeling

CFD analysis of a control model and an assembled anchor and IVC model was completed using ANSYS Fluent software (ANSYS 2022 R1). The control model refers to the IVC without anchor. The geometry was first meshed using structured hexahedral elements for both the anchor and the IVC. A grid size of 1.2 mm was selected. Local refinement was completed to maintain adequate mesh resolution. A mesh convergence study was completed, and 501,000 elements were used for model.

**Figure 7:**
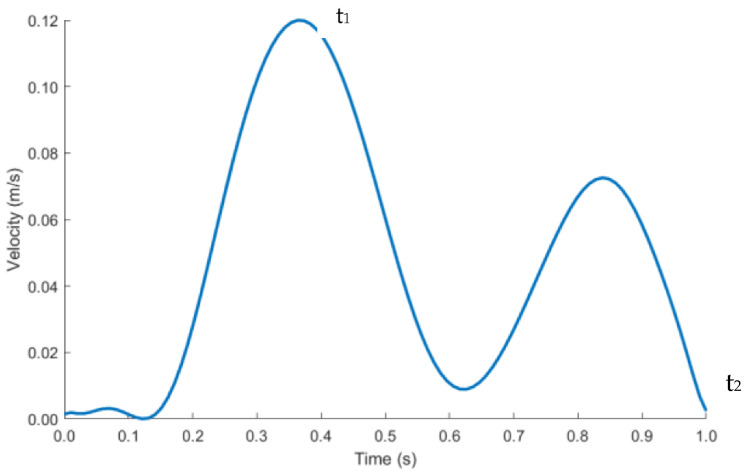
Pulsatile inlet velocity profile.

The length of the IVC and iliac veins were extended to 300 mm to allow enough length for blood flow to develop and for the results to be independent of the vein lengths. Velocity inlet boundary condition was imposed at the right and left iliac vein and pressure outlet at the IVC, labelled in Figure 6b. As blood flow is pulsatile in nature, a transient velocity inlet profile for a cardiac cycle was selected as shown in Figure 7. A user defined function (UDF) was written to define the blood flow during one cardiac cycle. The blood velocity function against time was taken from literature studies [46]. The performance of the anchor was analyzed at peak systolic (maximum blood velocity) t1 and end diastolic (minimum blood velocity) t2 time points during a cardiac cycle.

The pressure outlet was set as 0-gauge pressure. No-slip boundary conditions were applied to the walls of the geometry. The flow for the simulation was considered transient and incompressible. The calculated maximum Reynolds Number and Womersley Number in the IVC were 1500 and 9.6, respectively. This suggests that the blood in the IVC can be characterized as laminar and plug flow.

Blood can be modeled as Newtonian or non-Newtonian fluid [47]. Viscous stresses arising from the flowing motion of blood is directly proportional to the local strain rate in Newtonian blood flow. This is a simplistic approach to define the flow behavior of blood. Blood more closely mimics the properties of a non-Newtonian fluid. Viscous stresses of the blood are dependent on the strain rate varying throughout the cardiac cycle and geometry of the IVC. Studying the risk of blood clotting and flow stagnation is one of the aims of the CFD analysis. It has been deduced that modeling blood as a non-Newtonian fluid computes more accurate shear stress values [48,49]. The accuracy of assessing the aforementioned risks is directly dependent on the accuracy of wall shear stress outputs from the CFD analysis. Most of the literature on the use of Newtonian or non-Newtonian fluid pertains to arteries. However, it is assumed that the same principles apply to veins due to similar vessel diameters.

The relationship between non-Newtonian blood dynamic viscosity η and shear rate $\dot{\gamma }$ is defined by the widely used Carreau–Yasuda equation [50,51], demonstrating the shear-thinning nature of blood.

(1) $$\eta \left(\dot{\gamma }\right)=\eta_{\infty }+\left(\eta_{0}-\eta_{\infty }\right){\left(1+{\left(\lambda \dot{\gamma }\right)}^{a}\right)}^{\left(\frac{n-1}{a}\right)}$$

The following constants defined by Boyd et al. [51] were used: $\eta_{0}$ = 0.0035 Pa·s, λ = 8.2 s, a = 0.64 and n = 0.2128. Blood density ρ was taken as 1060 kg/m^3^.

### 2.6. Haemodynamic Parameters

Blood flow through the anchor disturbs flow causing flow separation, resulting in blood tapering inwards distal to the anchor. This induces changes in the wall shear stress (WSS) in the anchor region, which can be evaluated through time averaged wall shear stress (TAWSS), oscillatory shear index (OSI) and relative residence time (RRT). These haemodynamic parameters are indicators of blood stagnation leading to blood clotting or thrombosis. If the haemodynamic parameters are above the critical ranges, the endothelial cells of the IVC adopt an atherosclerotic profile. The critical range is as follows: TAWSS < 0.4 Pa, OSI > 0.3 and RRT > 10 Pa^−1^ [51].

WSS is a vector quantity. TAWSS measures the magnitude component of the vector quantity. Low TAWSS values are indicators of low blood velocities. OSI quantifies the directional component of the vector quantity. It ranges between 0 (no change in direction of blood flow) and 0.5 (complete reversal of the blood flow direction, 180° deflection of the WSS vector). High OSI suggests that the blood is in oscillating state with a higher risk of blood clotting due to formation of secondary flow zones. High RRT reflects the residence time of blood particles stagnant at one location, as compared with blood particles in other locations of the IVC.

Equations (2)–(4) were used to calculate the haemodynamic parameters. Here, *T* is the time period for one cardiac cycle. WSS and *t* are the instantaneous wall shear stress and time stamp of the cardiac cycle.

TAWSS

(2) $$\mathrm{TAWSS}=\frac{1}{T}\int_{0}^{T}\left|\mathrm{WSS}\right|\;dt$$

OSI

(3) $$\mathrm{OSI}=\frac{1}{2}\left(1-\frac{\left|\int_{0}^{T}\mathrm{WSS}\;dt\;\right|}{\int_{0}^{T}\left|\mathrm{WSS}\right|\;dt}\right)$$

RRT

(4) $$\mathrm{RRT}=\frac{1}{\left(1-2*\mathrm{OSI}\right)*\mathrm{WSS}}$$

WSS magnitude and x, y, and z values were exported from ANSYS and post-processed in MATLAB (R2021B) to calculate TAWSS, OSI and RRT.

## 3. Results

### 3.1. Anchor Deployment and Compression Tests

The 10-strut and 5-strut anchors were subjected to manual compression tests, which simulate the vena cava collapse during an inspiration-expiration cycle. The compression tests were divided in three categories that simulated a 25%, 50%, and 100% collapse. This means that the IVC was compressed manually at 100% (full collapse), 50% (half collapse), and 25% (one-fourth collapse).

**Figure 8:**
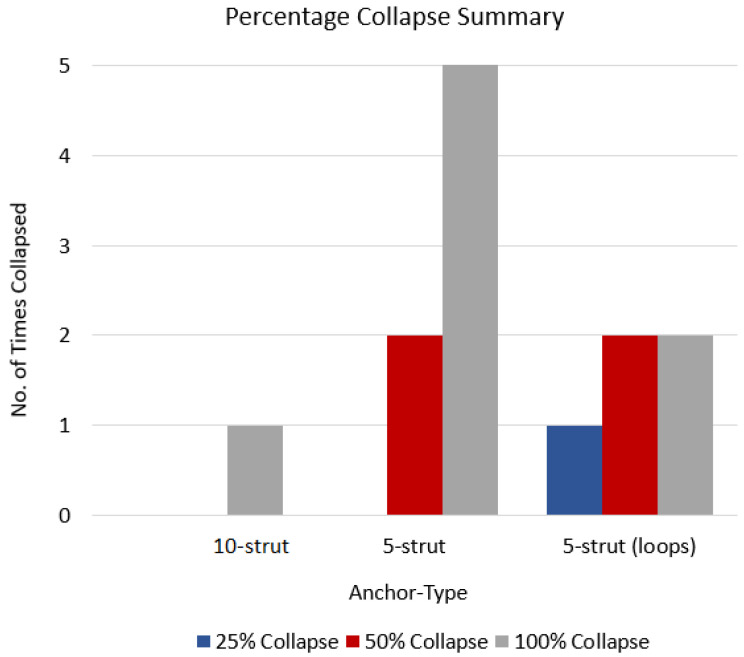
Anchor type vs. no. of times each anchor collapsed.

Five compression attempts in each category (each at 25%, 50%, and 100% per anchor design) resulted in anchor dislodgements, and these were represented as anchor type versus the number of times each anchor collapsed at 25%, 50%, and 100% (Figure 8). The 10-strut anchor collapsed only once at 100% due to strut-to-strut overlap. This is anticipated as there are numerous struts compactly arranged in a circular fashion. However, the movement or compression in relation to this design helped release the anchors thus resulting in recovery of original shape. The 5-strut anchor collapsed all attempts at 100% and twice at 50%. This design is less stable and moved along the length of the vein. The 5-strut with loops dislodged once at 25% and twice at 50% and 100%. Nevertheless, this design is stable when compared with the same version without loops and provides some resistance to dislodgement; it is still susceptible to migration due to fewer contact points with the vessel.

The 5-strut versions are not suitable to withstand the vena cava collapsibility and possess a higher risk of migrating to a zone of larger diameter and/or blocking renal or other veins in proximity. The 10-strut anchor has the ability to resist migrating forces simulated by manual compression. Additionally, the more the contact points with the vessel, the more stable the design.

### 3.2. CFD Analysis

The control (IVC without anchor) model was compared to the anchor model in CFD, which allowed analyzing the differences in fluid behavior due to the anchor.

#### 3.2.1. Velocity Profiles

**Figure 9:**
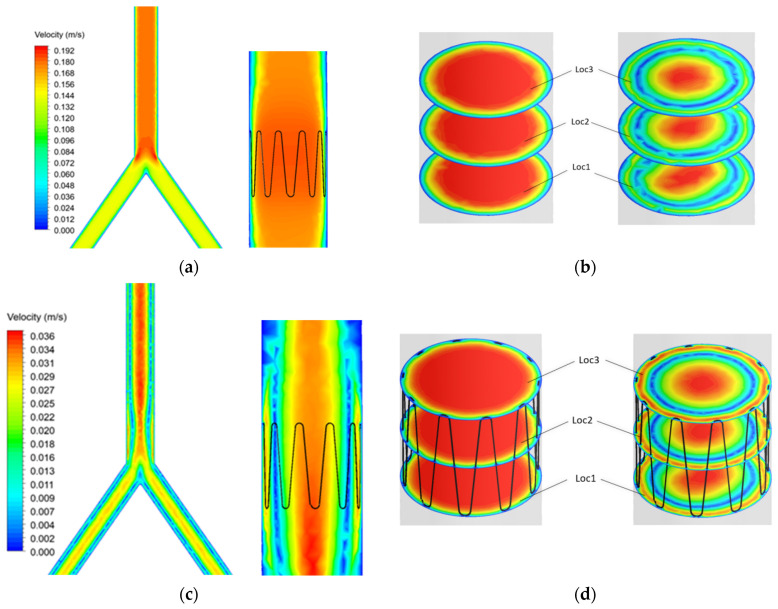
Control and anchor model at t_1_ (**a**) axial and (**b**) cross sectional velocity. Control and anchor model at t_2_ (**c**) axial and (**d**) cross sectional velocity.

Blood flow profiles were analyzed at time points t1 and t2 (Figure 7) during the cardiac cycle. A sectional plane was drawn along the axial length of the IVC model and velocity contour plotted (Figure 9a–c). Cross-sectional velocity profiles at three locations throughout the length of the anchor, proximal (Loc1), midpoint (Loc2), and distal (Loc3) of the anchor are illustrated in Figure 9b–d.

The results showed that blood velocity is much higher during systole (t_1_) as compared with diastole (t_2_). Flow separation can be seen at the bifurcation point. However, this does not affect blood flow in the anchor as the anchor is placed further into the IVC.

**Figure 10:**
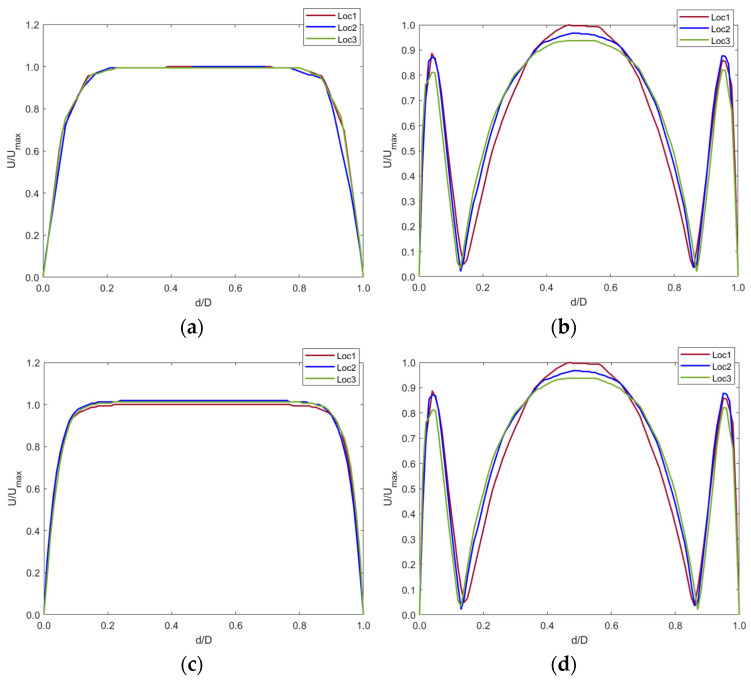
Velocity profiles at (**a**) t_1_ and (**b**) t_2_ for the control model and (**c**) t_1_ and (**d**) t_2_ for the implanted model.

The contour plots in Figure 9 were graphed using a normalized index. The plots show the blood velocity U against the diameter d of the IVC (Figure 10).

The graphs show that the anchor did not significantly affect the blood velocity as the graphs are identical for both the control and implanted model.

**Figure 11:**
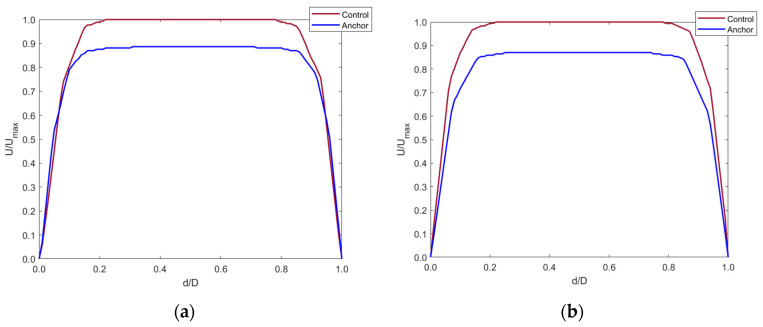

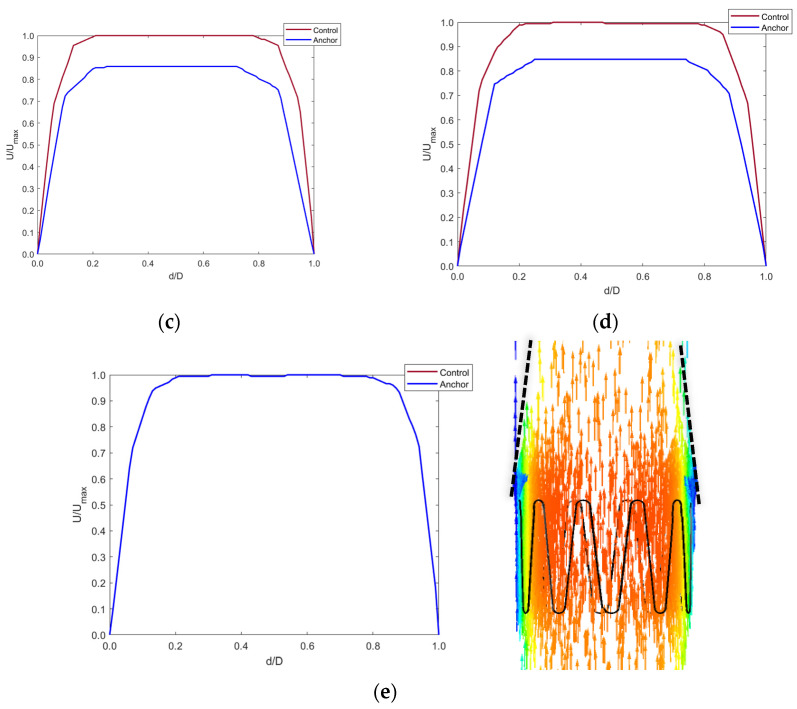
Axial velocity profiles distal to the anchor end at (**a**) 5 mm, (**b**) 10 mm, (**c**) 15 mm, (**d**) 20 mm, and (**e**) 40 mm in the IVC along with contour plot showing the tapering effect of blood flow distal to the anchor up-to 40 mm.

Any changes in the blood flow distal to the anchor were compared with the control model with the axis normalized (Figure 11). Cross-sectional planes were taken at five locations distal to the anchor. Velocity was plotted along the centerline of the cross-section planes. The y-axis shows the fraction of velocity in the implanted model as compared to the control model. The x-axis shows the normalized diameter of the IVC.

Figure 11a–d shows that blood velocity reduced by around 90% of the control model blood velocity. This reduction in velocity was seen up to 20 mm distal to the anchor end. This is due to the flow separation caused by the apex of the struts. The blood layer momentarily detached from the IVC wall distal to the anchor, creating a tapering effect of the blood flow. At around 40 mm distal to the anchor, the blood velocity increased to that in the control model as the effects of flow separation cease when blood flows along the IVC length. This is shown by Figure 11e.

#### 3.2.2. Haemodynamic Parameters

**Figure 12:**
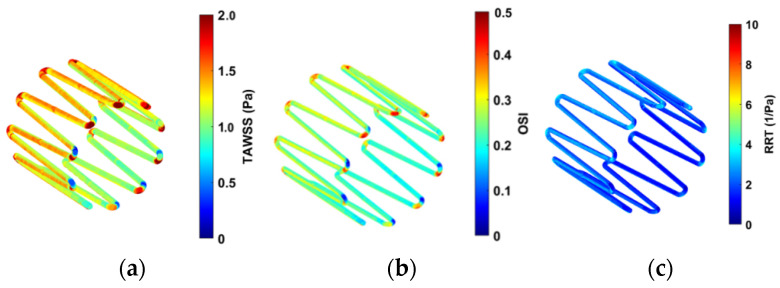
Contour plots for (**a**) TAWSS, (**b**) OSI, and (**c**) RRT.

Contour plots of the haemodynamic parameters (TAWSS, OSI, and RRT) were computed using MATLAB, as shown in Figure 12.

High OSI values are seen at the edges of the strut in Figure 12b. This is expected as the edges of the anchor can change the direction of the blood velocity vectors flowing across it. Lower OSI is observed along the length of the struts. Figure 12c shows low RRT values on the anchor surface.

**Figure 13:**
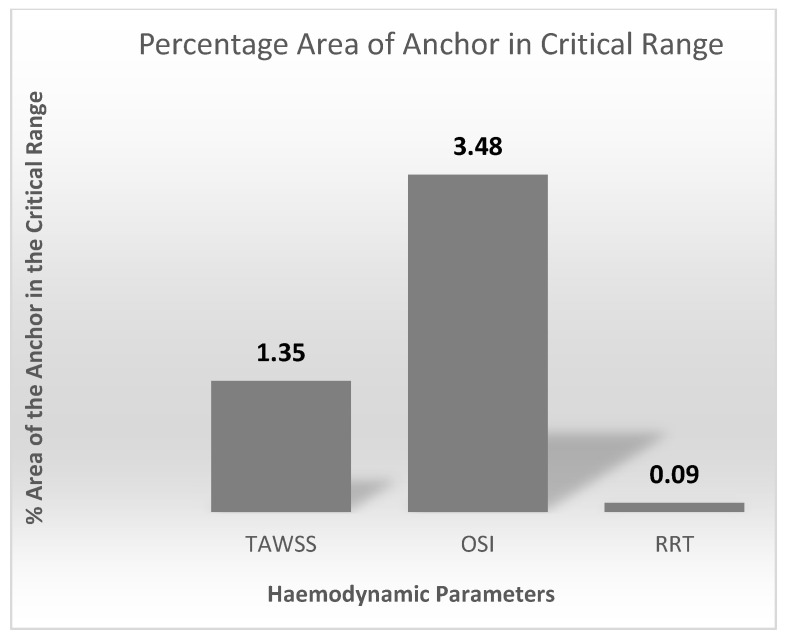
Percentage area of the anchor above the TAWSS, OSI, and RRT critical ranges.

Figure 13 quantifies the contour plots in Figure 12. This is represented as percentage area of the anchor above the critical ranges.

## 4. Discussion

This study presented the first steps in designing anchors for an active sensing implant suitable for the inferior vena cava for central venous pressure measurement. The anchors designed were feasible to implant in a silicone vasculature of vena cava (United Biologics, Inc., Irvine, CA, USA) and provided good anchorage at the target site. The 10-strut anchor design is more stable than the two other 5-strut designs. Although all anchor designs passed the basic feasibility tests on the bench, only the 10-strut anchor could withstand migration forces induced by manual compression attempts that simulated vena cava collapse. The methods applied to evaluate the anchor designs allowed identifying challenges in relation to the mode of deployment depending on the complexity and size of the anchor and selection of an optimal size delivery system. The double-introducer push-rod concept proved to be an effective delivery system to deploy the redefined anchors. The 14 Fr size (Cook Medical) was a good fit to load the implant, while an 18 Fr size (Cook Medical) was ideal for use as an outer introducer to guide to the target location.

The results from CFD analysis provided an understanding of the blood flow through the length of the anchor. Comparison between the control and anchor model allowed identifying any changes in blood flow due to the presence of the anchor. The Reynolds and Womersley values calculated suggested that the blood flow in the IVC is in the laminar regime and adopts a plug flow profile. There was no significant change in the blood flow due to the presence of the anchor. The edge of the anchor caused some flow separation, which led to the flow stream distal to the anchor to detach slightly from the IVC walls. This ‘tapering’ effect of blood stream occurred up to 40 mm distal to the anchor end. After this, the blood resumed a plug flow profile as any disturbances in blood due to flow separation ceases. A small increase in the percentage surface area of the anchor in the critical range was observed. This is expected as the edge of the anchor causes a disturbance in the blood flow. The critical range refers to a zone of low blood velocity and higher OSI, WSS, and RRT. The percentage surface area of the anchor in the critical range was minimum and less significant. Clinically, this would mean that future patients could be associated with a low risk of blood clotting, blood stagnation, or recirculation zones from IVC implants.

According to physicians, the implant procedure in IVC should take no more than fifteen minutes. Nonetheless, there is a high risk of device migration in the IVC as evident from the lessons learnt from IVC filters. Other complications include IVC occlusion, vessel penetration, filter embolization, and access site thrombus. Moreover, the pelvic movements also have an influence on the implants in the IVC region that contribute to device migration or dislodgement [39,40,41,42]. Nevertheless, the IVC could be considered a potential site for an implant mainly due to its feasibility and ease of implantation, less procedure time and minimal associated complications during implantation. Moreover, the implantation associated risks versus post-implant associated complications need to be explored in the IVC.

Haemodynamic sensors in the IVC combined with a safe anchoring system could help in establishing long-term continuous central venous pressure waveforms. Current sensor technologies such as CardioMEMS [20] (Abbott) and Cordella^TM^ implant [52] (Endotronix Inc., Chicago, IL, USA) focus on remote monitoring of PAP. Their trials specifically focus on NYHA class 3 HF patients regardless of ejection fraction, who are at a higher risk of rehospitalization from HF. Clinicians have been using these technologies as a means to providing remote care to their HF patients by keeping them out of the hospital [20,52]. There is a rapid interest in the cardiac, pulmonary, and renal communities around the possibility of similar benefits accruing from therapy guided by frequent, longitudinal measurements of Central Venous Pressure (CVP) [16,45]. CVP waveforms can provide more information about the inspiratory effort, compliance of the right ventricle, and the possibility of a cardiac tamponade [53]. Information about the effect of CVP measurement on the clinical outcomes is limited and should be explored extensively. High CVP values are associated with the risk of developing new or persistent acute kidney injury [54]. It is critical to understand how CVP measurement influences a clinician’s decision to manage and treat HF.

Several studies have highlighted the importance of IVC anchoring and attempts to address failed anchoring using T-fasteners and medical snares [55,56]. The retrieval of temporary IVC filters and repositioning of stents (Z-stents) is possible using medical snares. Snares have a three-looped structure made from nitinol wires to grasp foreign body objects easily. Nevertheless, the use of snares to fix stent malposition has yielded unsuccessful outcomes resulting in entanglement of the anchoring hooks of Z-stent with the snare. This further prevented the retrieval process of the stent, as there was a risk of anchoring hooks lacerating the right heart or the IVC [56]. The problems addressed in these studies arise from the complexity of device designs and the physiology of IVC. A greater outward radial force is the key to prevent device migration in the vena caval region. However, this design optimization demands for the use of a much larger introducer [56], which limits physicians from using or deploying designs that do not comply with the standard catheter systems routinely used in clinical practice. It is essential to consider these factors for designing a safe and optimal anchoring system in the IVC.

Future directions for designing an ideal venous implant (stents) focus on maintaining a balance between flexibility and radial force. Flexibility enables the stent to conform to the shape of the vein without kinking because of pelvic movements and decreasing the cross-sectional area [57]. Stents used in the arterial system are quite rigid, which leads to non-conformity of the stent and straightening of the vein. Rigidity is also common in venous stents placed at the iliocaval junction extending towards the infrarenal IVC. This often requires stent overlap to cover the length of the disease. The overlap could result in increased rigidity and decreased conformity within the IVC [57]. This would clinically affect the acute and long-term outcomes for patients resulting, in decreased blood flow and progression of cardiovascular disease. These parameters are crucial to the design of an anchor in the IVC; however, the application requires placement of the sensor-anchor above the iliocaval junction. Therefore, rigidity is less of a concern in the IVC as compared with flexibility and radial force. Future directions for the design optimization of the 10-strut anchor could focus on increasing the length of the anchor and thickness to improve the ORF and flexibility. An ideal design for an anchor in the IVC may involve incorporating a part of the IVC filter including hooks, and a part of stent including longer struts arranged in an open-cell fashion. The hooks will ensure there is no migration within the IVC, whereas the open cell design with a longer length and optimal thickness will improve overall flexibility and ORF. The complexity arising from a combination of these parameters need to be assessed for safety and feasibility of implantation in-vitro and in-vivo.

## 5. Limitations

The 10-strut anchor is an optimal design and feasible for implantation in-vitro, however it needs to undergo a series of ORF, strength and fatigue tests to validate safety and efficacy of implantation in-vivo. A second limitation is the addition of hooks or barbs to the anchor design to improve the ORF and prevent risk of dislodgment to a zone of larger diameter. The addition of hooks demands for a more complex design of the anchor and involves a higher risk of penetrating the IVC wall over a certain period. Therefore, it requires several attempts to develop the ideal structure for a venous implant that would afford the best balance between the said design parameters.

The deployment mechanism using the introducer system can sometimes cause the anchor to spring radially within the IVC. This can result in anchor deployments slightly away (usually upwards) from the target location. To mitigate the risks involved with this deployment technique, we placed the introducer loaded with the anchor slightly above the iliac bifurcation and a few inches below the target location to achieve the desired orientation and deployment. The sensor body is facing laterally within the introducer so that the radial spring force results in a deployment with sensor body attached to the anterior wall of vessel. This could also help to prevent any tilts during anchor deployment.

The orientation of the sensor body can affect the measurements. If the sensor is located at the posterior wall of the vessel, the link distance (distance between the implant and the skin surface) increases and the accuracy of the measurements is compromised. The deployment technique employed in this study helped to identify challenges with orientation or positioning of the sensor within the IVC, required hand-push force to deploy the anchor, and its effects (tilts or migration) on the deployment accuracy.

The CFD simulation assumes that the IVC walls are rigid. A rigid wall model could overestimate wall shear stress and thus the haemodynamic parameters. This suggests that the estimated values might be an over prediction. Fluid Structure Interaction (FSI) modeling can be completed to incorporate changes in blood flow through the anchor due to the elasticity of the IVC walls. Further analysis of performance of the anchor should include patient specific IVC models to create realistic 3D anatomical maps of various IVC morphologies. This would provide an insight into designing more flexible, tortuous designs, accommodating irregularities in the IVC.

## 6. Conclusions

This study successfully demonstrated the design and deployment of an IVC anchor system in a bench test model. The bench test simulation provided useful insights into the design and fabrication, selection, and analysis of the anchor, while the CFD simulation provided an understanding of the haemodynamic changes around the anchor zone, as well as risk factors anticipated post implantation. However, substantial in-vivo experiments are required to validate the safety and accuracy of the IVC implant. The clinical value of this research focuses on an improved assessment of the potential value of CVP measurements in predicting and managing congestive heart failure that will drive specific diagnoses and treatment modalities. The ability to place a sensor technology in the vena cava could provide a simple and minimally invasive approach for HF patients.
